# Development of a gastroretentive delivery system for acyclovir by 3D printing technology and its *in vivo* pharmacokinetic evaluation in Beagle dogs

**DOI:** 10.1371/journal.pone.0216875

**Published:** 2019-05-15

**Authors:** Soyoung Shin, Tae Hwan Kim, Seok Won Jeong, Seung Eun Chung, Da Young Lee, Do-Hyung Kim, Beom Soo Shin

**Affiliations:** 1 College of Pharmacy, Wonkwang University, Iksan, Jeonbuk, Korea; 2 College of Pharmacy, Daegu Catholic University, Hayang-eup, Gyeongsan, Gyeongbuk, Korea; 3 School of Pharmacy, Sungkyunkwan University, Jangan-gu, Suwon, Gyeonggi-do, Korea; 4 KNOTUS Co., Ltd. Research center, Guri, Gyeonggi-do, Korea; Brown University, UNITED STATES

## Abstract

Gastroretentive (GR) systems are designed to prolong gastric residence time to allow sustained absorption and improve the oral bioavailability of drugs with a narrow absorption window in the upper part of the gastrointestinal tract. The present study aimed to develop a GR system for acyclovir using 3D printing technology and evaluate its *in vivo* pharmacokinetics after oral administration in Beagle dogs. The system consisted of a gastro-floating device, which can float in the gastric fluid, prepared by a fused deposition modeling 3D printer and conventional acyclovir sustained-release (SR) tablet. The acyclovir SR tablet was inserted to the floating device to allow sustained release of the drug in the stomach. The buoyancy and sustained-release property of the developed GR system were determined using an *in vitro* dissolution test, *in vivo* pharmacokinetic study, and abdominal X-ray imaging in Beagle dogs. The *in vivo* dissolution profiles of the GR system were also predicted based on the *in vivo* pharmacokinetic data using a population pharmacokinetic (POP-PK) model. In the dissolution test, the sustained-release characteristic of the GR system was identified with a time corresponding to 80% dissolution (T_80_) of 2.52 h. Following oral administration of the GR system, the time to reach the maximum concentration (T_max_) of acyclovir was significantly prolonged, whereas the maximum concentration (C_max_) decreased and the area under the curve increased compared with those obtained after the administration of immediate-release and SR tablets, indicating prolonged absorption. By X-ray imaging, we showed that the developed GR system stayed in the stomach for more than 12 h. The POP-PK model successfully described the observed plasma concentration-time data and predicted the *in vivo* biphasic dissolution profiles of the GR system, which was significantly different from the *in vitro* dissolution. The developed GR system could be applied to various drugs and had great prospects in the design and development of novel controlled-release formulations.

## Introduction

Gastroretentive (GR) systems are designed to retain drug formulations in the stomach for an extended period and increase the oral bioavailability of drugs that have limited absorption window in the upper small intestine. Drugs with low stability in the lower part of the gastrointestinal tract or drugs that act locally within the stomach can also benefit from GR systems [1, 2]. Since GR system was introduced almost three decades ago, various approaches have been applied to extend the gastric residence time of GR systems [1] including low-density (floating), high-density (sinking), expandable (swelling), and mucoadhesive systems. Innovative approaches, such as magnetic field-assisted gastro-retentive systems, plug-type swelling systems, floating systems with or without effervescence, have also been applied to prolong gastric retention time [3]. These systems have been utilized either by themselves or in combination with one another, and they have shown promising results *in vitro* [4]. However, compared to the promising *in vitro* results, the *in vivo* and commercialization successes of GR systems are not satisfactory [3]. Physiological conditions of the stomach and gastric retention and emptying time are highly variable. The main challenge is to maintain the drug delivery system in the stomach for a sufficient time until all the drugs are released at a predetermined rate in a dynamic physiological condition.

Acyclovir [9-(2-hydroxyethoxymethyl)guanine; Zovirax] is a widely used antiviral drug for treating infectious diseases, such as genital herpes, chicken pox, varicella zoster infections, and herpes keratitis caused by herpes simplex virus. Oral absorption of acyclovir is highly variable, with an average bioavailability of 20–26.7% in humans [5–7]. Moreover, due to its short half-life (t_1/2_) of approximately 2.5 h, repeated administration of high-dose acyclovir is usually required to achieve therapeutic efficacy. For example, the oral dose used to treat genital herpes is 200 mg five times daily for ten days. Therefore, various studies had been conducted to increase the bioavailability and decrease the dosing frequency of acyclovir by using alternative routes of administration, such as intravitreal, nasal, vaginal, and ocular drug delivery systems. Nevertheless, because the oral route remains the most preferred, oral delivery systems of acyclovir, including microemulsion [8, 9], gastric mucoadhesive sustained-release microsphere [10], inclusion complexes with cyclodextrins [11], and gastro-floating systems [4, 12, 13], have been extensively explored.

Acyclovir is known to be mainly absorbed in the upper part of the gastrointestinal tract [14, 15]. Reducing the delivery rate of acyclovir to its absorption site in the gut by repeated oral administrations of small amounts of acyclovir significantly improved its absorption [14]. In addition, it was reported that oral administration of a magnet embedded sustained-release (SR) tablet prolonged the gastric residence and improved the bioavailability of acyclovir in the presence of extracorporeal magnet [15]. These results indicate that acyclovir has an effective absorption window in the gastrointestinal tract. For drugs that have a narrow absorption window, reduced relative bioavailability of their SR tablets is often observed as their dissolution rate decrease, which is probably because the slowly released drug from the SR tablets may bypass the main absorption site, resulting in low overall absorption [16]. Thus, among several approaches in the design and development of novel formulations for acyclovir, gastroretentive (GR) drug delivery systems may be one of the most promising.

Three-dimensional (3D) printing technology is a layer-by-layer process to form 3D objects from digital designs and it enables on-demand fabrication of objects of almost any shape and size. Introduction of 3D printing to pharmaceuticals may cause a paradigm shift in design, manufacture, and use of drugs. In 2015, the US Food and Drug Administration (FDA) approved the first 3D-printed tablet, Spritam [17] which is now commercialized to treat epilepsy. Currently, 3D printing has been extensively explored and applied to produce oral solid dosage forms by printing multiple active pharmaceutical ingredients with different geometries and release characteristics [18–24].

In this study, through application of 3D printing technology, a GR system for acyclovir was developed for oral administration with sustained release, thereby maintaining plasma acyclovir concentration for an extended period. The main advantages of 3D printing technologies include its rapid prototyping, which is a versatile tool to develop novel pharmaceutical dosage formulations with various and complex geometry. Thus, we aimed to design a gastro-floating device by 3D printing technology and insert a sustained-release (SR) tablet of acyclovir to the device. The floating ability of the device was attributed to the air pockets inside the device, which was printed by a fused deposition modeling (FDM) 3D printer. The formulation of acyclovir inserted into the floating device allowed sustained release of the drug. Therefore, the final GR system for acyclovir could remain in the stomach and slowly release the drug for an extended period. The floating ability and sustained release property of the developed GR system were evaluated by *in vitro* dissolution test, *in vivo* X-ray imaging, and pharmacokinetic studies in Beagle dogs. Finally, the *in vivo* dissolution of the GR system was predicted by a population pharmacokinetic (POP-PK) modeling.

## Materials and methods

### Chemicals and reagents

Zovirax 200 mg was purchased from Dong-A Pharmaceutical Co., Ltd. (Seoul, Korea). Acyclovir, microcrystalline cellulose (Avicel), and anhydrous lactose were purchased from Whawon Pharm. Co. (Seoul, Korea). Hydroxypropyl methylcellulose (HPMC) 2208–100 cps was obtained from Shin-Etsu Chemical Co., Ltd. (Tokyo, Japan). Magnesium stearate was purchased from Faci Asia Pacific Pte Ltd. (Jurong Island, Singapore). Sodium hydroxide and sodium chloride were purchased from Samchun Chemical Co., Ltd. (Seoul, Korea). Baclofen, an internal standard for the liquid chromatography-tandem mass spectrometry (LC-MS/MS) assay, acetic acid, and formic acid were purchased from Sigma-Aldrich Co. (St. Louis, MO). Ethanol (HPLC grade), hydrochloric acid, and potassium dihydrogen phosphate were purchased from Merck Co. (Darmstadt, Germany). High-performance liquid chromatography (HPLC) grade acetonitrile and water were purchased from J.T. Baker Co. (Philipsburg, NJ). Polylactic acid filament was obtained from Raise3D, Inc. (Costa Mesa, CA).

### Formulation

SR tablets containing 100 mg of acyclovir were prepared by using HPMC as a drug release modifier. Lactose and Avicel were used as diluents. The composition of the acyclovir SR tablet was 100 mg of acyclovir (50%), 78 mg of Avicel (39%), 20 mg of HPMC2208-100 (10%), and 2 mg of magnesium stearate (1%). The formulation of the SR tablet was intended to achieve complete drug release during the residence of the final GR system in the stomach. SR granules were prepared by the wet granulation method. Acyclovir was mixed with diluents and kneaded with HPMC dissolved in ethanol, and then the dampened mixture was passed through a size-20 mesh screen. Next, the wet granules were dried in an oven at 60°C. The dried granules were again passed through a size-20 mesh screen, and then mixed with lubricant (1%). Then, the mixture was compressed at a force of 5 kN by a hydraulic tablet press (Carver, Inc., Wabash, IN) with a round-shaped punch (diameter: 9.1 mm). Since immediate-release (IR) reference tablet containing 100 mg of acyclovir is not commercially available, an IR tablet containing 100 mg of acyclovir was prepared by compressing the milled powder of Zovirax 200 mg.

### 3D printing of the gastro-floating device

**Fig 1:**
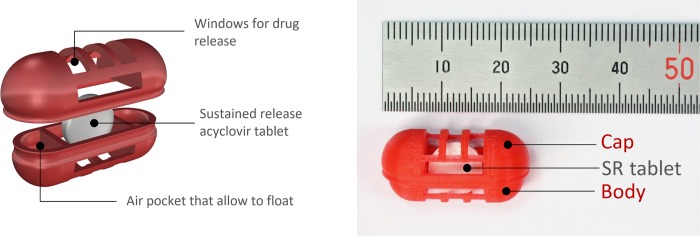
The structure of the gastroretentive (GR) system. The novel GR system composed of the 3D printed gastro-floating device and acyclovir sustained-release (SR) tablet inside the device.

The structure of the gastro-floating device was designed by the computer-aided design software, Rhino 5 (Robert McNeel & Associates, Seattle, WA). The oblong shape of the device consisted of two separated parts, the body and cap, and three compartments inside (Fig 1). The device was designed to have two closed compartments on the left and right as air pockets, and one compartment in the middle wherein a tablet can be inserted, when the body and cap are tightly locked. The central compartment was designed to have windows for drug release. Due to the nature of additive manufacturing, the hollow shape device could be produced by adding layer-upon-layer without sacrificial materials. Different sizes and shapes of the drug releasing windows were compared to evaluate their effects on the drug releasing rates from the inner SR tablet. The small sizes of drug releasing windows resulted in slow dissolution rates and incomplete drug release, which may be due to the limited movement of dissolution medium. As the sizes of drug releasing windows become bigger, drug release rates increased similar to that of intact SR tablet. The shape of the windows also affected the drug release properties. For example, from a capsular device with a large circular shape of the window, the inner SR tablet evacuated before it is completely dissolved. Therefore, the final gastro-floating device was designed to have sufficient sizes of windows for drug release and hold the inner SR tablet during until the drug dissolution is completed (Fig 1).

The gastro-floating devices were printed with a commercial polylactic acid filament using an FDM 3D printer (Raise3D N2, Raise3D, Inc.). Extruder and platform temperatures were set at 210°C and 65°C, respectively. After the devices were printed, an SR tablet of acyclovir was inserted into the center compartment of the body, and the cap was locked to form the final GR system (Fig 1).

### *In vitro* dissolution test

To evaluate the *in vitro* dissolution of acyclovir from the reference IR tablet, SR tablet, and GR system, the paddle method was employed by using a Distek Dissolution System 2500 coupled with an Evolution Dissolution Sampler 4300 (Distek, Inc., North Brunswick, NJ, USA). As a dissolution medium, 0.1 N HCl buffer (pH 1.2) was used, and the temperature was maintained at 37 ± 0.5°C. The stirring speed of the paddle was fixed at 50 and 100 rpm. The samples were collected at the predetermined time, and the medium was replaced with fresh medium. Collected samples were filtered through a 45-μm polyethylene syringe filter (Distek, Inc.) and immediately analyzed by the HPLC method.

### Pharmacokinetic study

Beagle dogs (16–18 months; 10.5–11.7 kg) were purchased from Orient Bio (Seongnam, Korea). The animal studies were approved by the ethics committee for the treatment of laboratory animals at KNOTUS Co., Ltd (KNOTUS IACUC 18-KE-230). For X-ray imaging, a fragment of barium sulfate (BaSO_4_) fiber was inserted into one of the air pockets of the device and the GR system was orally administered to the dogs. Abdominal X-ray images were captured at 0, 1, 2, 4, 8, 12, 24, and 48 h after oral administration of the GR system.

For the pharmacokinetic study, the dogs were randomly divided into three groups: groups administered the reference IR tablet (n = 5), SR tablet (n = 5), and GR system (n = 5). The dogs were administered the acyclovir formulations under fed condition. After overnight fasting, the animals were fed a canned diet (Cesar; Mars, Inc., McLean, VA, USA) and water 30 min before drug administration. The test formulations; acyclovir IR tablet, SR tablet, or GR system containing 100 mg of acyclovir was orally administered to Beagle dogs. The test formulation was placed over the base of the tongue and the mouth was closed. The dog’s neck was gently stroked to ensure that the dog swallowed the formulation, followed by administration of water 10 mL with a syringe. Blood samples (3 mL) were collected from the jugular vein and placed in a heparinized (5 IU/mL) tube at 0, 0.25, 0.5, 0.75, 1, 1.5, 2, 2.5, 3, 4, 5, 6, 8, 12, 24, 36, and 48 h following drug administration. Plasma samples were obtained by centrifugation of the collected blood at 4,000 × g at 4°C for 10 min, and then stored at -20°C until analysis. The physiological conditions of the animals were carefully monitored by normal physical examination during the study and no signs of changes in the physiological status or potential side effects including vomiting and pain were observed. After completion of the study, the animals were placed back into the animal housing facility.

### Drug analysis

#### HPLC

Acyclovir concentrations in the dissolution medium were determined by HPLC using a Waters Alliance 2695 coupled with the Waters photodiode array detector 2996 (Waters, Milford, MA, USA). Acyclovir was separated on a Synergi 4 μ Fusion-RP 80Å (150 × 2.00 mm, i.d., 4 micron; Phenomenex, Torrance, CA, USA). An isocratic solvent system consisting of 0.1% formic acid in water and acetonitrile (95:5, v/v) was used as the mobile phase with a flow rate of 0.3 mL/min. The column oven temperature was set at 40°C and the total run time was 4.0 min. The sample injection volume was 2 μL and acyclovir was detected at 251 nm. Working standard solutions for HPLC analyses were prepared by serial dilutions of the stock solution in the mobile phase at concentrations of 5, 10, 20, 50, 100, 200, and 250 μg/mL.

#### LC-MS/MS

Acyclovir concentrations in dog plasma were determined by LC-MS/MS, which comprised of an Agilent 6430 triple-quadrupole mass spectrometer coupled with an Agilent 1200 HPLC (Agilent Technologies, Santa Clara, CA, USA). Acyclovir was separated on a Zorbax SB-Aq column (100 × 2.1 mm, i.d., 3.5 μm; Agilent). An isocratic solvent system consisting of 0.1% formic acid in water and methanol (90:10, v/v) with a flow rate of 0.2 mL/min was used as the mobile phase. The column oven temperature was 40°C and the total run time was 9 min. The mass spectrometer was operated using electron spray ionization in the positive ion mode with mass transitions of m/z 226.1 → 152.1 for acyclovir and m/z 214.0 → 151.0 for baclofen. Plasma samples were prepared by the protein precipitation method using methanol. The lower limit of quantification (LLOQ) was 50 ng/mL, with a range of intra-and inter-day accuracy of 89.7–108.8% and precision within 11.0%.

### Non-compartmental analysis

The pharmacokinetic parameters of acyclovir were determined by non-compartmental analysis using the Phoenix WinNonlin software (Certara, L.P., Princeton, NJ, USA). These parameters included terminal half-life (t_1/2_), area under the plasma concentration-time curve from time zero to the last observation time point (AUC_all_) and to infinity (AUC_inf_), apparent clearance (CL/F), apparent volume of distribution (V_z_/F), and mean residence time (MRT). Maximum plasma concentration (C_max_) and time to reach C_max_ (T_max_) were obtained directly from the observed data. Relative bioavailability (F_rel_) was estimated as the dose-normalized AUC_inf_ ratio between the test and reference IR formulation.

### Prediction of *in vivo* dissolution profiles from the *in vivo* pharmacokinetic profiles

**Fig 2:**
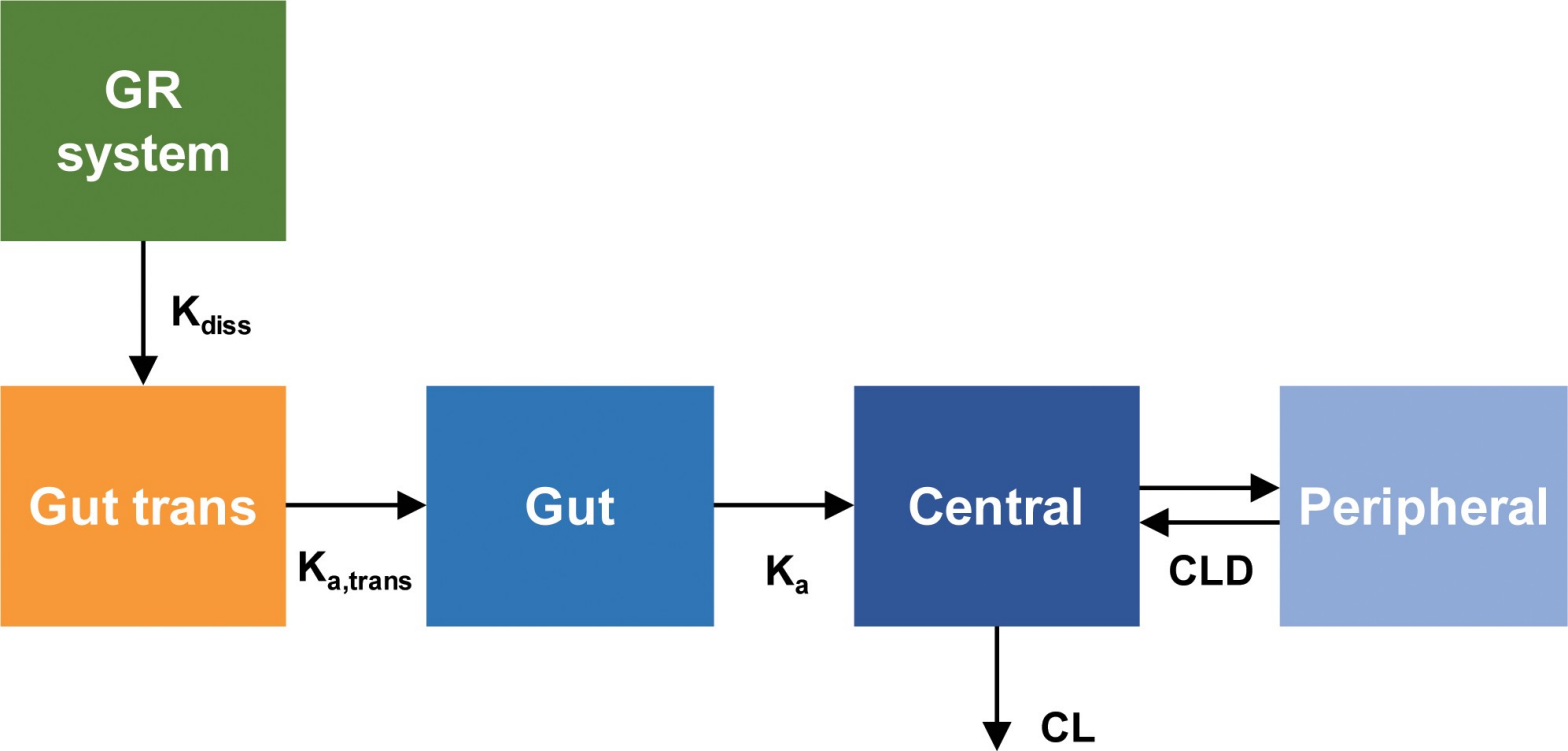
The pharmacokinetic model structure for the prediction of *in vivo* dissolution profile of acyclovir gastroretentive (GR) system. The population pharmacokinetic model of acyclovir to predict *in vivo* dissolution profile of GR system.

The *in vivo* dissolution profiles of the GR system were predicted from the plasma concentration-time data obtained after the oral administration of GR system by a population pharmacokinetic (POP-PK) modeling. The structural model for the pharmacokinetics of acyclovir GR system is depicted in Fig 2.

The *in vivo* dissolution of acyclovir from GR system was described by a first order rate process. The differential equation for the amount of acyclovir in the GR system was given by:

$$\frac{{\mathrm{dX}}_{\mathrm{GR}\;\mathrm{system}}}{\mathrm{dt}}=-K_{\mathrm{diss}}(t)\cdot X_{\mathrm{GR}\;\mathrm{system}}$$ (Eq 1)

where X_GR system_ is the amount of drug in the GR system and K_diss_(t) represents the dissolution rate constant which was permitted to change over time in order to reflect the atypical dissolution profile of the GR system. K_diss_(t) was described by using the Hill-type equation:

$$K_{\mathrm{diss}}(t)=K_{\mathrm{diss}}(0)\cdot \left[1+{\mathrm{Diss}}_{\max}\cdot \frac{{{(\mathrm{time}‐T}_{\mathrm{lag}})}^{10}}{T_{\mathrm{change}50}{}^{10}+{{(\mathrm{time}‐T}_{\mathrm{lag}})}^{10}}\right]$$ (Eq 2)

where K_diss_(0) represents the initial dissolution rate constant and Diss_max_ is the maximal change of K_diss_ over time. A positive Diss_max_ leads to the increase of K_diss_ over time while a negative Diss_max_ represents the decrease of K_diss_ over time. T_lag_ is the lag time before the initiation of SR tablet dissolution from the GR system and T_change50_ is the time associated with a half-maximal change of K_diss_ during the increase of dissolution rate, i.e., reaching the maximum dissolution of SR tablet in GR system after complete swelling. The Hill coefficient was fixed at 10 to support estimation. Since IR tablet was assumed to be dissolved immediately in the gut, the dissolution model for IR tablet was not necessary. To describe the initial delayed absorption, a transit compartment was added before the gut compartment and IR tablet was directly introduced to the transit compartment. The differential equation for the amount of acyclovir in the transit compartment (X_Gut-trans_) was written as:

$$\frac{{\mathrm{dX}}_{\mathrm{Gut}‐\mathrm{trans}}}{\mathrm{dt}}=K_{\mathrm{diss}}(t)\cdot F_{\mathrm{rel}}\cdot X_{\mathrm{GRsystem}}-K_{a,\;\mathrm{trans}}\cdot X_{\mathrm{Gut}‐\mathrm{trans}}$$ (Eq 3)

F_rel_ is the relative bioavailability of the GR system compared with the IR tablet and K_a,trans_ is a first order rate constant to describe the transfer of the dissolved acyclovir from the transit compartment to the gut compartment. The absorption of the acyclovir from the gut compartment into the central compartment was then described by the first-order rate constant K_a_. The differential equation for the amount of acyclovir in the gut compartment *in vivo* (X_Gut_) was given by:

$$\frac{{\mathrm{dX}}_{\mathrm{Gut}}}{\mathrm{dt}}=K_{a,\mathrm{trans}}\cdot X_{\mathrm{Gut}‐\mathrm{trans}}-K_{a}\cdot X_{\mathrm{Gut}}$$ (Eq 4)

The systemic disposition of acyclovir was described by the two-compartment model. The acyclovir in the central compartment was assumed to be distributed to the peripheral compartment and eliminated from the central compartment. The differential equations for the amounts of acyclovir in the central (X_c_) and peripheral compartments (X_p_) were:

$$\frac{{\mathrm{dX}}_{C}}{\mathrm{dt}}=K_{a}\cdot X_{\mathrm{Gut}}-{\mathrm{CL}}_{D}\cdot C_{1}+{\mathrm{CL}}_{D}\cdot C_{2}-\mathrm{CL}\cdot C_{1}$$ (Eq 5)

$$\frac{{\mathrm{dX}}_{P}}{\mathrm{dt}}={\mathrm{CL}}_{D}\cdot C_{1}-{\mathrm{CL}}_{D}\cdot C_{2}$$ (Eq 6)

C_1_ and C_2_ represent acyclovir concentrations in the central and peripheral compartment, respectively. CL_D_ is the distribution clearance to the peripheral compartment, and CL is the systemic clearance.

The observed plasma concentration-time data were fitted to the POP-PK model using the Monte Carlo Parametric Expectation Maximization (MC-PEM) algorithm in the parallelized S-ADAPT software (version 1.57). An importance sampling MC-PEM method (pmethod = 4 in S-ADAPT) was used for population parameter estimation. Between-subject variability (BSV) was estimated using an exponential parameter variability model. Simulations were performed by using the Berkeley Madonna software (version 8.3.18). The predictive performance of the POP-PK model was evaluated by visual predictive checks as well as the prediction error (%PE). The %PE was calculated as follows:

$$\%\mathrm{PE}=\frac{\left|\mathrm{Predicted}-\mathrm{Observed}\right|}{\mathrm{Observed}}\times 100$$ (Eq 7)

### Statistical analysis

Obtained data were analyzed with the one-way analysis of variance followed by the Tukey’s post-hoc test for comparisons among more than two means of unpaired data. Data were expressed as the mean ± standard deviation (SD). The level of statistical significance was set at p<0.05 (SPSS Statistics 17.0; SPSS Inc., Chicago, IL, USA).

## Results

### The *in vitro* floating ability of the gastroretentive system

**Fig 3:**
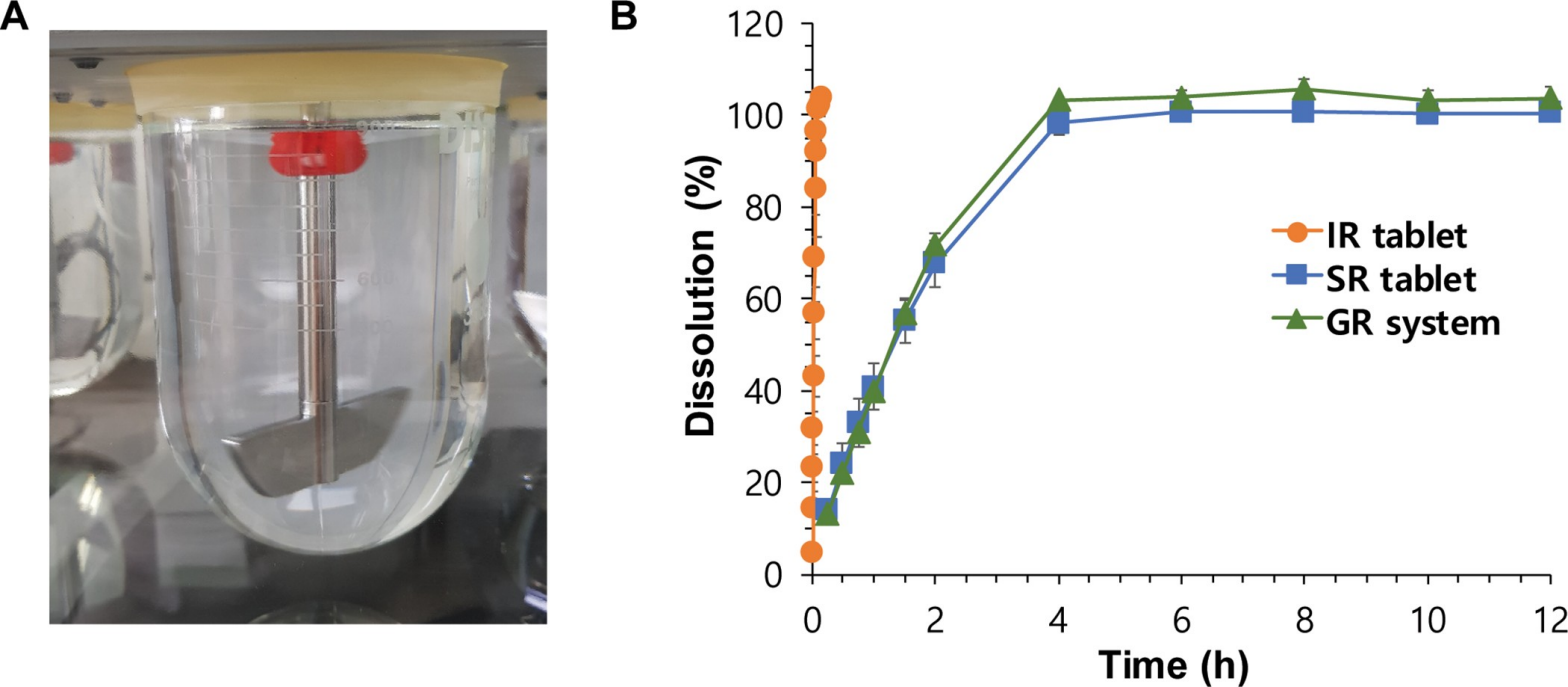
*In vitro* evaluation of the gastroretentive (GR) system. *In vitro* (A) floating ability and (B) dissolution profiles of the GR system compared to the immediate-release (IR) and sustained-release (SR) tablets. Data are presented mean ± SD (n = 4).

The *in vitro* floating ability of the GR systems was examined before their *in vitro* drug-release test. IR and SR tablets immediately sank to the bottom of the dissolution medium. However, the GR systems, which contained air pocket compartments, were buoyant for more than 24 h in the pH 1.2 medium (Fig 3A). Moreover, the devices also remained rigid. By comparing GR systems with different structures of drug-releasing windows, we showed that the shape and size of the drug-releasing windows did not affect the *in vitro* floating ability of the GR system.

### The *in vitro* dissolution of the gastroretentive system

The *in vitro* dissolution of the GR systems was evaluated by the paddle method. Despite their comparable floating capabilities, different GR systems with differently shaped and sized drug-releasing windows showed different drug-release profiles. Finally, the gastro-floating device that had five rectangular-shaped windows (Fig 1) was selected for use in the final GR system. The area of opened drug-releasing windows was 60% of the total surface area of the SR tablet containing part. Fig 3B shows the *in vitro* drug-release profiles of the IR (reference) tablet, SR tablet, and optimized GR system in a pH 1.2 medium at 100 rpm. The IR tablet showed complete drug-release within 5 min, whereas the SR tablet and GR system showed significantly slower drug-release than IR tablet. The time corresponding to 80% dissolution (T_80_) of the IR tablet, SR tablet, and GR system was 0.09 ± 0.01 h, 2.78 ± 0.26 h, and 2.52 ± 0.12 h, respectively.

### The *in vivo* floating ability of the gastroretentive system

**Fig 4:**
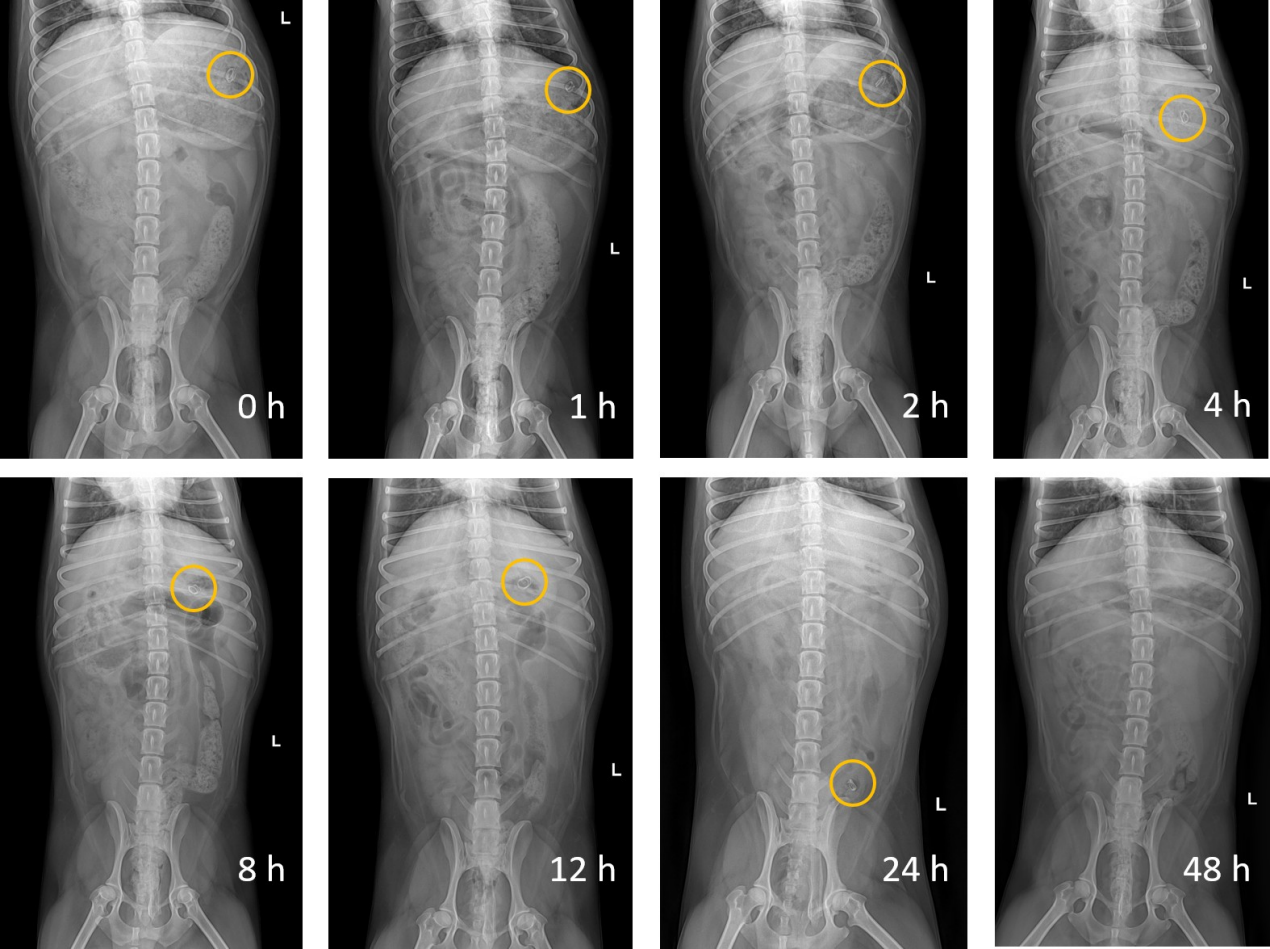
Abdominal X-ray images of the gastroretentive (GR) system in a Beagle dog. Abdominal X-ray images indicating the positions of the GR system in the gastrointestinal tract of a Beagle dog following oral administration for 24 h. The circles represent the GR system in the Beagle dog.

The *in vivo* floating ability of the GR system was examined after oral administration to Beagle dogs. Fig 4 shows X-ray images of the BaSO_4_-labeled GR system, which remained in the stomach of the dogs. The GR system was observed to remain in the stomach for more than 12 h, present in the small intestine at 24 h, and disappear at 48 h after oral administration.

### The *in vivo* pharmacokinetics of the gastroretentive system

**Fig 5:**
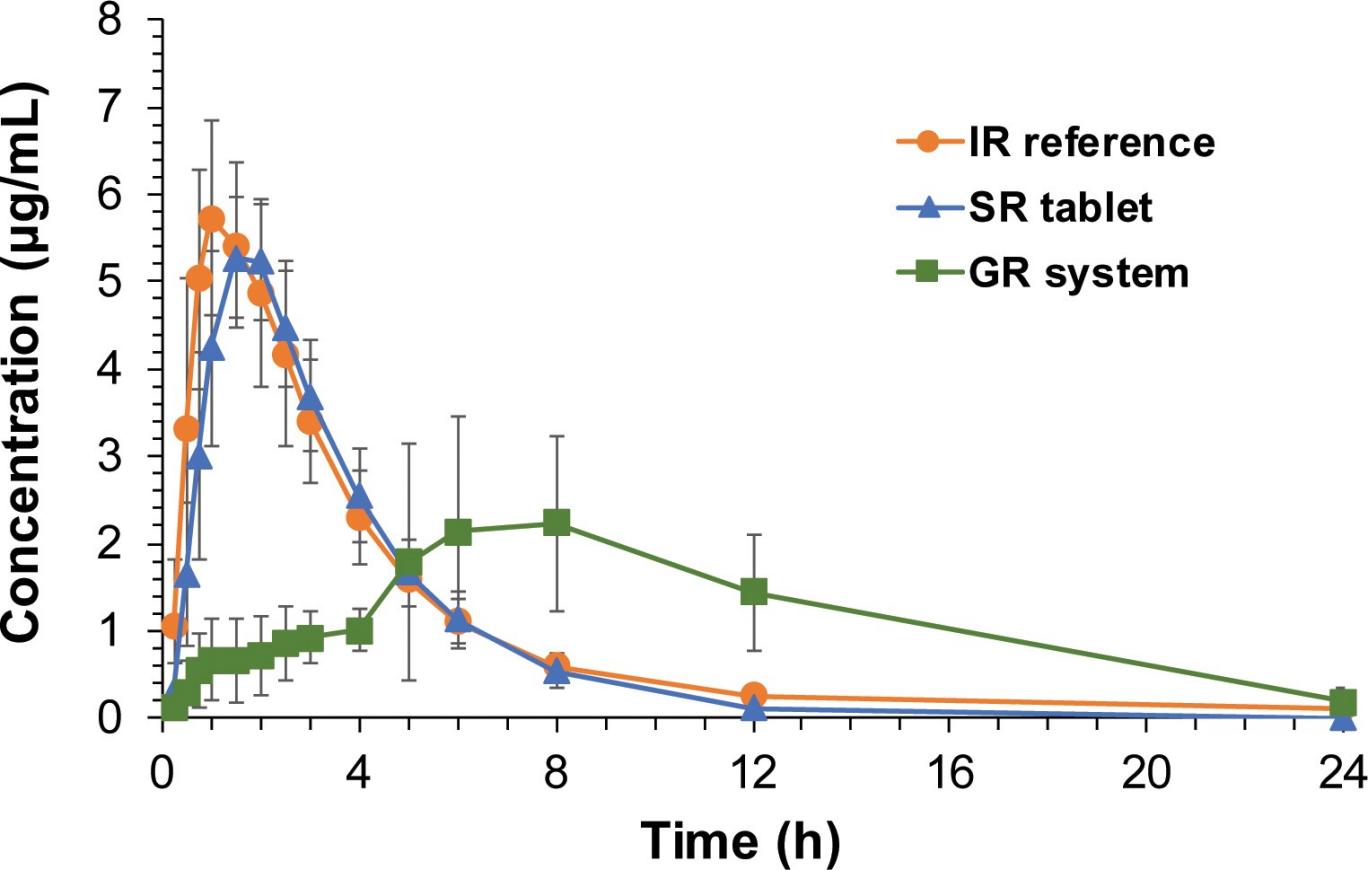
*In vivo* pharmacokinetics of the gastroretentive (GR) system in Beagle dogs. Plasma concentration vs. time profiles of acyclovir following oral administration of the immediate-release (IR), sustained-release (SR), or GR system of acyclovir in Beagle dogs. Data are presented mean ± SD (n = 5).

The mean plasma concentration-time profiles of acyclovir observed after oral administration of the IR tablet, SR tablet, and GR system to the dogs are depicted in Fig 5. The pharmacokinetic parameters of the non-compartmental analysis are summarized in Table 1. Following oral administration of the IR tablet, acyclovir was rapidly absorbed and achieved C_max_ within 1.5 h, and plasma acyclovir concentrations declined with a t_1/2_ of 3.22 ± 1.45 h after that. Compared to the IR tablet, the SR tablet presented delayed T_max_. SR tablets showed slightly shorter t_1/2_ and lower C_max_ and AUC values than those of IR tablets, but the differences were not statistically significant. On the contrary, the GR systems of acyclovir showed significantly prolonged T_max_ and MRT, as well as lower C_max_ values, compared to those of IR and SR tablets. The AUC values of the GR system were higher than those of the IR and SR tablets. Therefore, F_rel_ of the SR tablet and GR system was estimated to be 89.84% and 122.67%, respectively (Table 1).

**Table 1:** Non-compartmental pharmacokinetic parameters of acyclovir after oral administrations of immediate release (IR) tablet, sustained release (SR) tablet, or gastroretentive (GR) system in Beagle dogs (n = 5).

Parameters	IR tablet (n = 5)	SR tablet (n = 5)	GR system (n = 5)
Dose (mg)	100	100	100
t_1/2_ (h)	3.22 ± 1.45	1.80 ± 0.17	4.47 ± 1.43
T_max_ (h)	1.10 ± 0.22	1.70 ± 0.27*	9.20 ± 2.68*
C_max_ (μg/mL)	5.84 ± 1.18	5.61 ± 0.38	2.80 ± 1.12*
AUC_all_ (μg·h/mL)	22.56 ± 3.05	20.84 ± 2.20	28.06 ± 6.05
AUC_inf_ (μg·h/mL)	23.56 ± 2.95	21.16 ± 2.36	28.90 ± 5.52
V_z_/F (L)	20.44 ± 11.08	12.32 ± 0.92	23.64 ± 10.82
MRT (h)	4.27 ± 1.23	3.49 ± 0.45	10.39 ± 2.13*
CL/F (mL/min)	71.56 ± 8.12	79.55 ± 8.97	59.30 ± 10.73
F_rel_ (%)	-	89.84	122.67

*, p<0.05 vs. IR reference tablet.

### Prediction of *in vivo* dissolution profiles from the *in vivo* pharmacokinetic profiles

**Fig 6:**
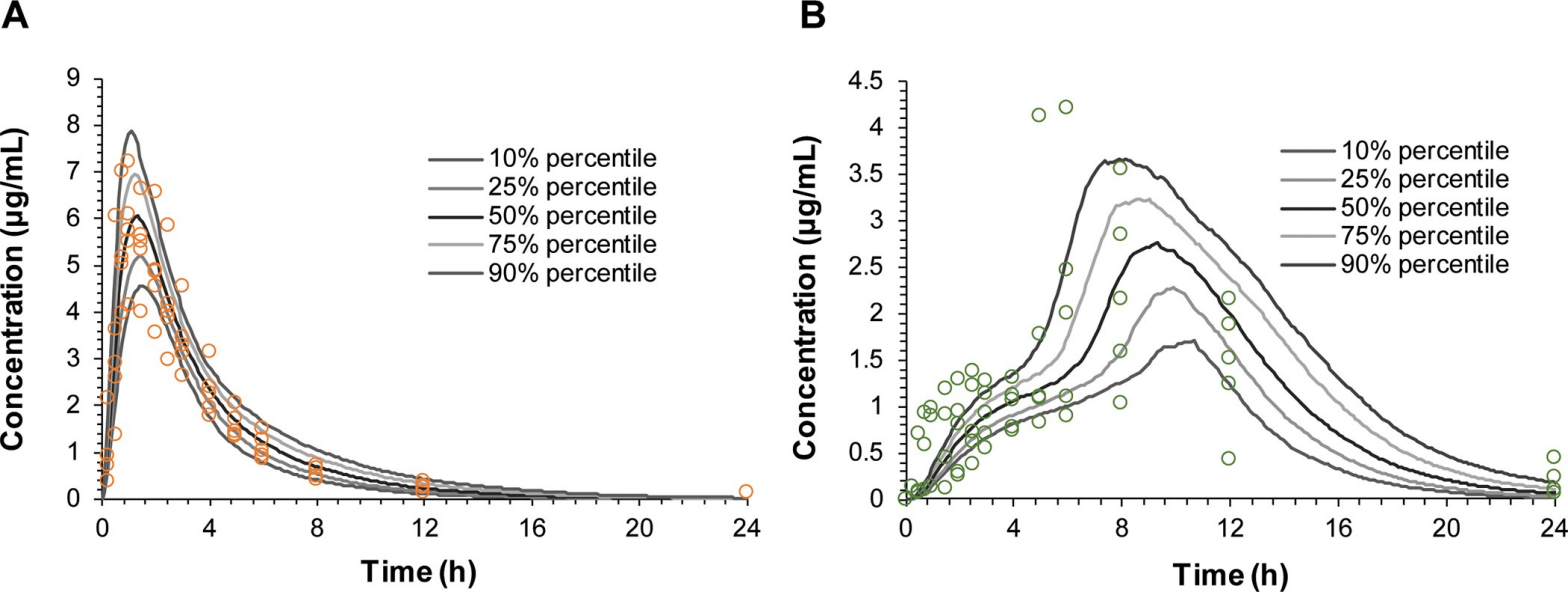
Visual predictive check plots of the population pharmacokinetic (POP-PK) model. Visual predictive check plots for (A) immediate-release (IR) tablet and (B) gastroretentive (GR) system for the validation of the final POP-PK model to predict *in vivo* dissolution profiles. The open circles represent the observed data, and the lines represent the predicted profiles obtained from final POP-PK model.

The POP-PK model was developed to estimate the *in vivo* dissolution of SR tablet in the GR system based on the plasma concentration-time profiles. The POP-PK model consisted of two disposition compartments, and drug dissolution and absorption were separately described by the time-dependent first-order kinetics and the first-order kinetics, respectively. By changing the first-order rate constant of dissolution (K_diss_) over time, the biphasic absorption, i.e., the initial slow absorption followed by the rapid absorption, could be described. Fig 6 shows the observed and the POP-PK model-predicted plasma concentration-time profiles for IR reference tablet and GR system. The overall plasma concentration-time data were well described by the POP-PK model. The prediction error (PE%) of the C_max_ and AUC compared to the observed values were 5.87% and 1.35%, respectively for IR tablet and 11.8% and 7.09%, respectively for GR system. The goodness-of-fit plots shown in S1 Fig also showed that the POP-PK model predicted plasma concentrations and the observed concentrations were close to the line of identity without significant bias, indicating that the POP-PK model was able to characterize the observed data reasonably well.

**Fig 7:**
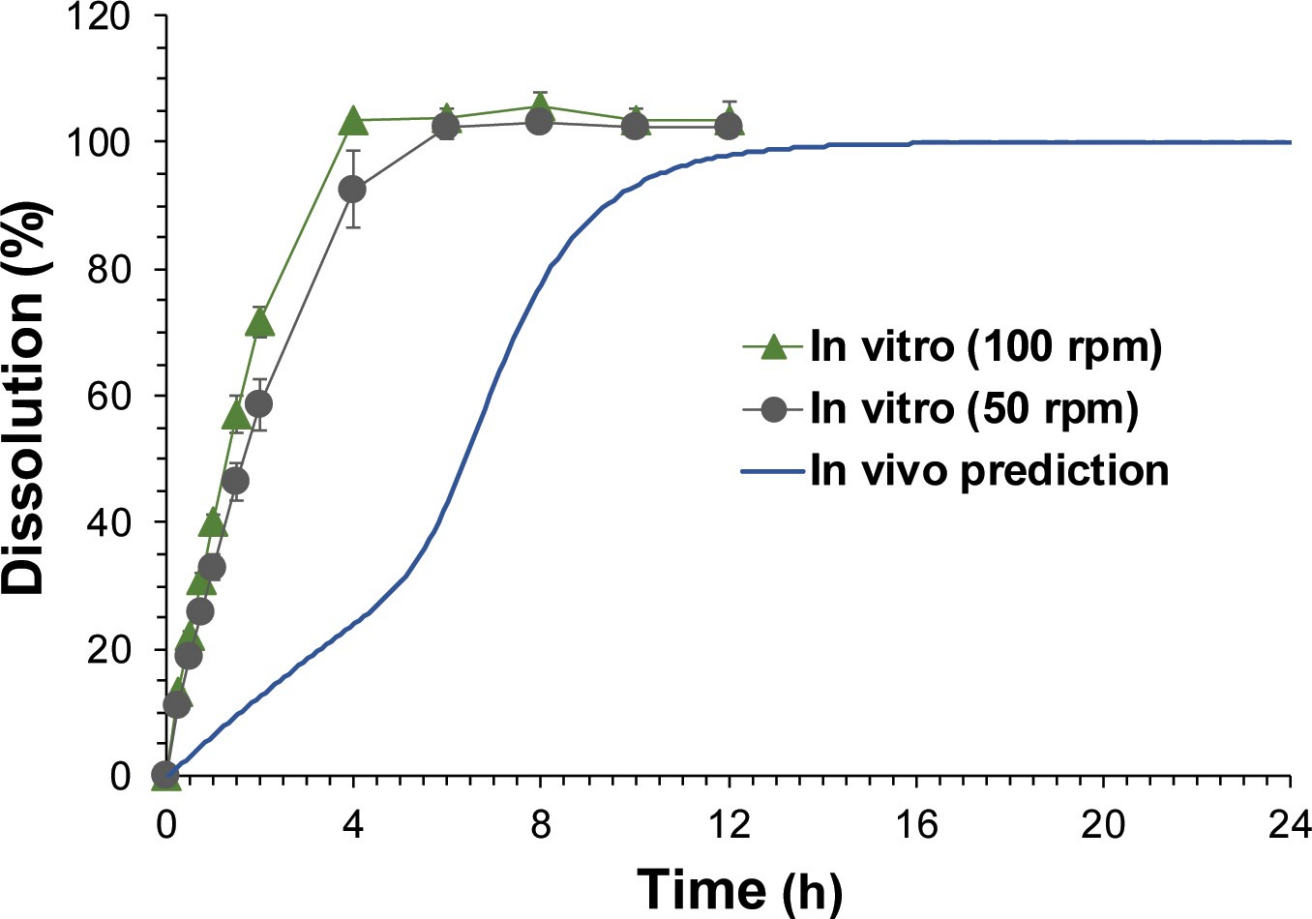
*In vivo* dissolution profiles of acyclovir gastroretentive (GR) system predicted by the population pharmacokinetic (POP-PK) model. The POP-PK model-predicted and observed dissolution profiles of acyclovir GR system. Symbols represent *in vitro* dissolution profile obtained by the paddle method with the paddle stirring speed at 50 and 100 rpm, and the solid line represents *in vivo* prediction obtained by POP-PK model prediction.

The *in vivo* dissolution profiles extracted from the plasma concentration-time profiles by the POP-PK model compared with the observed *in vitro* dissolution profiles obtained with the paddle speed at 50 and 100 rpm are presented in Fig 7. The *in vitro* dissolution profiles obtained with the paddle speed of 50 and 100 rpm were comparable with T_80_ of 3.28 ± 0.34 h and 2.52 ± 0.12 h, respectively. However, the predicted *in vivo* dissolution rates were significantly slower (T_80_ = 8.22 h) than the observed *in vitro* dissolution rates regardless of the paddle speed. Moreover, the shapes of the dissolution profiles were different between the *in vitro* and *in vivo* dissolution profiles. While the *in vitro* dissolution profiles presented a single-phase drug release, the *in vivo* dissolution was predicted to be biphasic, which showed an initial slow release followed by a rapid release. The POP-PK parameter estimates for GR system are presented in Table 2. The POP-PK model indicated that the dissolution is initiated after a lag time (T_lag_) of 1.13 min from the GR system and the dissolution rate increases to the maximum dissolution at T_change50_ of 6.15 h after the oral administration of the GR system. The estimated F_rel_ for GR system was 119% which is comparable with the observation.

**Table 2:** Population pharmacokinetic parameter estimates of acyclovir immediate release (IR) tablet and gastroretentive (GR) system.

Parameters	Mean (SE%)	BSV (SE%)
V_1_ (L)	8.83 (12%)	0.315 (56.2%)
V_2_ (L)	3.47 (10.5%)	0.11 (342%)
CL (L/h)	4.57 (4.5%)	0.123 (50.6%)
CLD (L/h)	1.75 (4.1%)	0.0336 (170%)
T_lag_ (h)	0.0189 (55.1%)	0.18 (200%)
K_diss_(0) (h^-1^)	0.0683 (15.1%)	0.161 (124%)
T_change50_ (h)	6.15 (11.3%)	0.285 (51.4%)
Diss_max_	7.86 (16.1%)	0.256 (67.2%)
Hill	10 (fixed)	0 (fixed)
K_a,trans_ (h^-1^)	2.63 (7.6%)	0.267 (51.8%)
K_a_ (h^-1^)	2.68 (10%)	0.189 (54.1%)
F_rel_IR_	1 (fixed)	0.0234 (348%)
F_rel_GR_	1.19 (8.5%)	0.0404 (187%)

## Discussion

Acyclovir is a Biopharmaceutics Classification System (BCS) class III drug that has high solubility and low permeability and is reported mainly absorbed in the upper part of the gastrointestinal tract [14, 15]. The presence of an absorption window may become a hurdle to sustain plasma drug concentrations and increase oral bioavailability because of the fast gastric emptying time. Therefore, GR formulation coupled with the extended drug release property may be the best alternative way to increase the oral bioavailability and prolong drug plasma drug concentrations for drugs with absorption window such as acyclovir.

In this study, a novel GR system of acyclovir was developed by combining a 3D-printed gastro-floating device with an acyclovir SR tablet. The major advantage of the present GR system is that its floating ability and drug-releasing property are independent of one another. The effectiveness of other conventional floating systems mostly depends on the balance between drug loading and polymer effect of its release profile [1]. Thus, the buoyancy of the system may decrease during drug release, leading to the premature evacuation of the system before drug release is complete. However, the present GR system had independently controlled floating and drug-releasing abilities, and its drug release did not affect its buoyancy and vice versa. Therefore, the excellent buoyancy of the system was maintained independently during drug release. In addition, the present GR system provided immediate buoyancy in the stomach owing to the air entrapped in the device. Other floating systems, such as gas-generating systems, usually have a lag time before they float [25, 26], which may result in premature evacuation of the dosage forms through the pyloric sphincter. Moreover, the present GR system can be applied to many other drugs by simply combining the gastro-floating device with any conventional SR tablet. Although the present study utilized acyclovir as a model drug, this approach could be applied to prolong the gastric residence time of other drugs, thereby improving their oral bioavailability and therapeutic efficacy.

3D printing technology has unique advantages over conventional manufacturing and is expected to bring a paradigm shift in pharmaceuticals. Thus, 3D printing has been extensively explored and applied to produce novel oral solid dosage forms with different geometries and release characteristics [27–31]. However, these approaches mainly rely on printing the drug-containing polymer filaments layer by layer to form a final drug product with different shapes and release characteristics for controlled release. Instead of printing a drug-containing filament to form a final drug product, this study utilized 3D printing technology as a tool to design and develop a capsular device having a gastro-floating capability and combined it with a conventional tablet. Since the floating ability was attributed to the structure of the capsular device, it was critical to design and compare various prototypes of the devices with different shapes and inner structures to achieve the optimal floating ability and drug release properties. However, with conventional manufacturing process, such as plastic molding, creating each prototype for the optimization process is costly and time-consuming. Therefore, the final gastro-floating device was optimized through testing of various prototypes with different structures by utilizing the advantage of rapid prototyping of 3D printing technology [32]. With 3D printing, various prototypes of gastro-floating device with different structures could be efficiently designed and manufactured, which can save a significant amount of time, money, and effort.

The final GR system that combined a 3D-printed gastro-floating device and an acyclovir SR tablet achieved the prolonged plasma drug concentration profiles and improved bioavailability compared to the IR tablet. In contrast, the conventional SR tablet of acyclovir by itself did not achieve sustained-release characteristics in the plasma drug concentration profiles and failed to improve oral bioavailability (Fig 5 and Table 1), despite its similar sustained *in vitro* drug release rate to that of the GR system (Fig 3). The pharmacokinetic differences between GR system and intact SR tablet may attribute to the prolonged residence of GR system in the stomach. Owing to the gastro-floating ability of the capsular device, GR system remained in the stomach and continuously released the drug to the absorption site, resulting in the sustained plasma drug concentration profiles and improved bioavailability.

The plasma drug concentration profiles following oral administration of the final GR system were further examined by POP-PK modeling to estimate the *in vivo* drug release from the GR system. The POP-PK modeling indicated that *in vivo* dissolution profiles of GR system was significantly different from the *in vitro* dissolution profiles. The predicted *in vivo* dissolution of GR system was significantly slower compared to the *in vitro* dissolution and showed clear biphasic profiles (Fig 7). Even with the slower paddle stirring speed at 50 rpm, the *in vitro* dissolution was still significantly faster than the predicted *in vivo* dissolution. Moreover, the biphasic dissolution profiles could not be obtained by the *in vitro* dissolution test. The discrepancy between *in vitro* and *in vivo* dissolution profiles may attribute to the various confounding factors present in the *in vivo* gastrointestinal tract, including the presence of food and viscosity of the gastric fluid. Even though the drug-releasing windows of the floating device have been designed large enough for free movement of water, the swelling of the SR tablet may be inhibited by the GR device in the gastrointestinal tract, which was represented by the initial slower drug release. After complete swelling, the drug release may be facilitated with the increased dissolution rate, resulting in the biphasic dissolution profile. This atypical *in vivo* performance of the formulation is hard to be predicted by the *in vitro* test and should be evaluated by the *in vivo* pharmacokinetic studies.

There have been several reports available to control drug-release profiles by combining a 3D-printed device and a conventional formulation, such as pulsatile release capsular device [33], sustained-release scaffold [34], and floating tablet-in-device systems [35]. However, their drug-releasing characteristics were only evaluated *in vitro* and their *in vivo* pharmacokinetics have not been reported. Since the *in vivo* drug-release profiles of the GR system may be significantly deviated from the *in vitro* dissolution profiles in the dissolution tester, it is critical to evaluate the *in vivo* pharmacokinetics during the development process for the formulations that combines a conventional tablet and a drug release controlling device. To best of our knowledge, this is the first study to report the *in vivo* pharmacokinetics of the 3D-printed capsular device. The potential reasons for the difference between the in vitro and in vivo drug release from the capsular devices may be worthy of further studies. More studies are also required to examine the safety of the GR system and the use of other completely biocompatible materials to produce of the capsular device.

## Conclusions

In summary, we have successfully showed the potential of a gastro-floating device combined with a conventional SR tablet of acyclovir to reduce the dosing frequency and improve the oral bioavailability of acyclovir. 3D printing technology allowed simple and efficient production of various prototypes of the device, as well as optimization of its structure, to achieve prolonged gastric residence. The extended gastric residence of GR system was monitored by X-ray images, and the prolonged plasma drug concentration profiles with the increased bioavailability were proven by the *in vivo* pharmacokinetic study. This approach may be useful for designing novel controlled release formulations by combining functional device and conventional formulations.

## Supporting information

S1 Fig Goodness-of-fit plots for the final population pharmacokinetic (POP-PK) model. (A) Plot of observed acyclovir plasma concentrations obtained after oral administration of immediate release (IR) tablet (n = 5) and gastroretentive (GR) system (n = 5) to Beagle dogs vs. individual predictions. (B) Plot of observations vs. population predictions. Open circles represent the observations and the line is the line of identity.
(TIF)
