# Supplementary figures and images for: Development of a gastroretentive delivery system for acyclovir by 3D printing technology and its in vivo pharmacokinetic evaluation in Beagle dogs
Source: PLoS One. 2019 May 15;14(5):e0216875. doi: 10.1371/journal.pone.0216875 (PMC6519832; doi:10.1371/journal.pone.0216875)

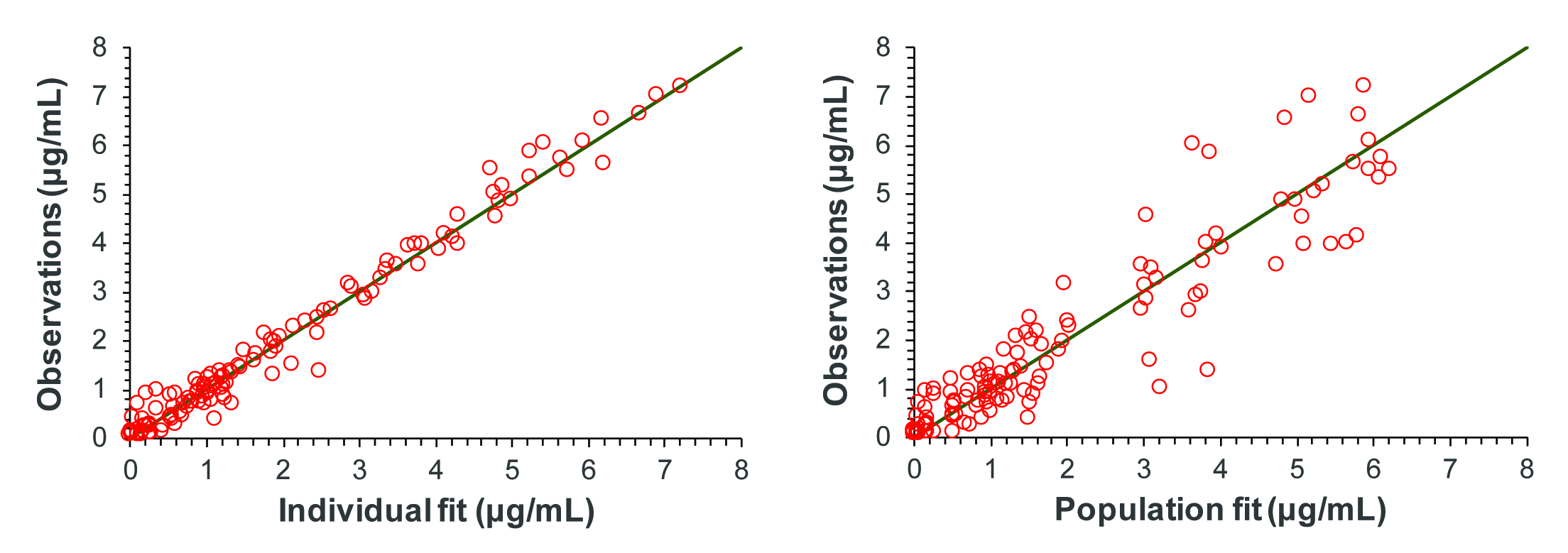

Supplement: S1 Fig — (A) Plot of observed acyclovir plasma concentrations obtained after oral administration of immediate release (IR) tablet (n = 5) and gastroretentive (GR) system (n = 5) to Beagle dogs vs. individual predictions. (B) Plot of observations vs. population predictions. Open circles represent the observations and the line is the line of identity. (TIF) [file pone.0216875.s001.tif]
